# Ultrasensitive DNA hypermethylation detection using plasma for early detection of NSCLC: a study in Chinese patients with very small nodules

**DOI:** 10.1186/s13148-020-00828-2

**Published:** 2020-03-05

**Authors:** Chen Chen, Xiaojie Huang, Wei Yin, Muyun Peng, Fang Wu, Xia Wu, Jingqun Tang, Mingjiu Chen, Xiang Wang, Alicia Hulbert, Malcolm V. Brock, Wenliang Liu, James G. Herman, Fenglei Yu

**Affiliations:** 1 Department of Thoracic Surgery, The Second Xiangya Hospital, Central South University, Changsha, Hunan People’s Republic of China; 2 Department of Cardiovascular Surgery, The Second Xiangya Hospital, Central South University, Changsha, Hunan People’s Republic of China; 3 Department of Oncology, The Second Xiangya Hospital, Central South University, Changsha, Hunan People’s Republic of China; 4 Department of Pathology, The Second Xiangya Hospital, Central South University, Changsha, Hunan People’s Republic of China; 5 Department of Surgery, University of Illinois at Chicago School of Medicine, Chicago, IL USA; 6 Department of Surgery, The Sidney Kimmel Cancer Center, Johns Hopkins University School of Medicine, Baltimore, MD USA; 7 UPMC Hillman Cancer Center, Department of Medicine, University of Pittsburgh School of Medicine, Pittsburgh, PA USA

**Keywords:** Lung cancer, Biomarker, DNA methylation, Plasma, Early detection

## Abstract

**Purpose:**

We had previously developed highly sensitive DNA methylation detection to diagnose lung cancer in patients with pulmonary nodules. To validate this approach and determine clinical utility in Chinese patients with indeterminate pulmonary nodules, we assessed the diagnostic accuracy for early stage lung cancer in plasma samples.

**Experimental design:**

Patients with CT-detected small lung nodules (diameter ≤ 3.0 cm) were included. Cases (*n* = 163) had staged IA or IB non-small cell lung cancer (NSCLC), while controls (*n* = 83) had non-cancerous lesions. Promoter methylation of eight lung cancer-specific genes (CDO1, TAC1, SOX17, HOXA7, HOXA9, GATA4, GATA5, and PAX5) was detected using nanoparticle-based DNA extraction (MOB) followed by qMSP.

**Results:**

Methylation detection for CDO1, TAC1, SOX17, and HOXA7 in plasma was significantly higher in cases compared with the benign group (*p* < 0.001). The sensitivity and specificity for lung cancer diagnosis using individual gene was 41–69% and 49–82%. A three-gene combination of the best individual genes has sensitivity and specificity of 90% and 71%, with area under the receiver operating curve (AUC) of 0.88, (95% CI 0.84–0.93). Furthermore, three-gene combinations detected even the smallest lung nodules, with the combination of CDO1, SOX17, and HOXA7 having the overall best performance, while the combination of CDO1, TAC1, and SOX17 was best in tumor sizes less than 1.0 cm.

**Conclusions:**

Using modified MOB-qMSP, high sensitivity and specificity, for the detection of circulating tumor DNA was obtained for early stage NSCLC. This strategy has great potential to identify patients at high risk and improve the diagnosis of lung cancer at an earlier stage.

**Graphical Abstract:**

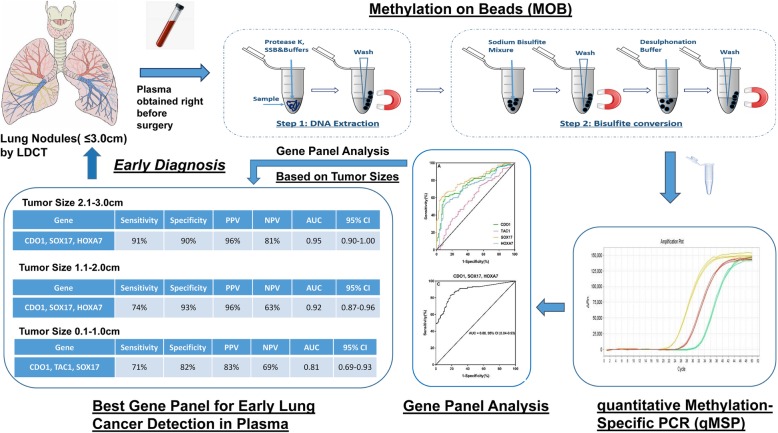

## Introduction

Lung cancer remains the leading cause of cancer-related death in the world [1, 2]. The increasing adoption of lung cancer screening has resulted in a rapidly growing number of patients with small lung nodules requiring management. Most of these nodules are benign but are hard to distinguish from small lung cancers. Thus, the benefits of low-dose computed tomography (CT) screening have been limited due to its low positive predictive value and high false positive rates [3–5]. Distinguishing small malignant nodules in CT scan from benign ones is particularly challenging because of their ambiguous radiographic characteristics [6, 7]. There are intense efforts to improve nodule management by identifying molecular biomarkers, including gene mutation [8–10], DNA methylation [11, 12], circulating tumor cells [10], and cancer-specific auto-antibodies [13], that could improve the diagnostic accuracy for detection of early stage of lung cancer.

Circulating tumor DNA (ctDNA), which refers to DNA released from lysed or apoptotic tumor cells and circulating freely in blood, is a promising approach for the detection of very early stage of lung cancer [14, 15]. Most previous studies focused on identifying particular gene mutations in ctDNA from lung cancer patients, such as EGFR, p53, and KRAS, as these are frequently mutated in lung cancers and some have implications in targeted therapies [8]. However, the diagnostic sensitivity for these targets is sharply decreased if the tumors were small or lack these mutations [16]. Methylation of cytosine in CpG islands silences hundreds of genes that are involved in the initiation and progression of lung cancer. Several studies have reported cancer-specific DNA methylation changes detectable in plasma, sputum, saliva, and pleural effusions from the patients with lung cancer [9, 11, 17].

Our previous case-control study reported that, using MOB-qMSP, an ultrasensitive DNA methylation detection approach using superparamagnetic nano-beads followed by quantitative methylation-specific real-time PCR, highly prevalent lung cancer-specific gene methylation could be detected in the plasma and sputum from patients with early-stage lung cancer [18–20]. In the present study, we optimized MOB-qMSP and validated its diagnostic utility in a Chinese patient cohort of indeterminate pulmonary nodules, all 3 cm or less in size.

## Materials and methods

### Study population

From December 2016 to April 2018, 345 patients with small indeterminate lung nodules (< 3 cm on CT scan) that were suspected to be non-small cell lung cancer (NSCLC) were included in this study. Patients who received any pretreatment therapy, including chemotherapy or radiotherapy, or had a history of malignancy were not included. All patients received curative-intent resection. All blood samples were obtained prior to surgery and were immediately processed to isolate plasma. Paired tumor samples were collected immediately after the tumor was removed and stored at − 80 °C. All patients with pathologically confirmed malignant lesions were staged according to the revised TNM guidelines classification criteria [21]. Patients with NSCLC were included as cancer group, those with histologically benign lesions as the control group. Plasma samples of 20 healthy volunteers (clinical characteristics provided in Supplemental Table S1) were also obtained as a normal group. Nodule size was obtained from the pathologic report. A summary of clinical and pathological characteristics of patients and healthy volunteers included in this study are presented in Table 1 and Supplemental Table S1.

**Table 1:** Clinical characteristics of the patients

Patient characteristics	Cancer group (*n* = 163)	Control group (*n* = 83)	*p* value
Age (year, mean ± SD)	58.79 ± 9.11	52.45 ± 7.27	0.01
Gender (%)			
Male	84 (51.5%)	52 (62.7%)	0.32
Female	79 (48.5%)	31 (37.3%)	
Stage no. (%)			
T1N0	102 (62.6%)	N/A	N/A
T2N0	61 (37.4%)	N/A	
Histologic characteristics no. (%)			
Adenocarcinoma	139 (85.3%)	N/A	N/A
Squamous-cell	22 (13.5%)	N/A	
NOS	2 (1.2%)	N/A	
Granuloma	N/A	35 (42.2%)	
Hamartoma	N/A	10 (12.0%)	
Inflammation	N/A	29 (34.9%)	
Fungal infection	N/A	2 (2.4%)	
Other benign	N/A	7 (8.5%)	
Nodule size (cm, mean ± SD)	1.78 ± 0.67	1.64 ± 0.73	0.33
0–1.0 cm	28 (17.2%)	22 (26.5%)	0.22
1.1–2.0 cm	92 (56.4%)	43 (51.8%)	
2.1–3.0 cm	43 (26.4%)	18 (21.7%)	
Pack-year (IQR)	38 (10–150)	35 (0.75–90)	0.57
COPD no. (%)	2 (1.2%)	2 (2.4%)	0.49
FEV1 (L, mean ± SD)	2.32 ± 0.79	2.65 ± 0.70	0.29
FVC (L, mean ± SD)	3.00 ± 1.02	3.42 ± 0.86	0.23
FEV1/FVC (%, mean ± SD)	73.52 ± 18.53	76.76 ± 14.31	0.11

*COPD*, chronic obstructive pulmonary disease; *FEV1*, forced expiratory volume in one second; *FVC*, forced vital capacity; *IQR*, interquartile range

Among the 345 patients, 34 patients were excluded due to failed ctDNA extraction mainly due to hemolysis or insufficient ctDNA yield. In this study, we used β-Actin (ACTB) as an internal control, with a CT value of ACTB more than 34 indicated insufficient ctDNA yield [18]. Additionally, pathology review revealed 3 cases of small cell carcinoma, 5 carcinoid, 4 unclassified carcinomas, and 53 patients with regional or mediastinal lymph node metastasis, and these patients were excluded from the analysis. In total, 163 patients in the cancer group with early-stage node-negative tumors (T_1-2_N_0_M_0_) and 83 patients in the control group with benign lung nodules had samples adequate for analysis. This study was approved by the Human Ethics Committee of the Second Xiangya Hospital, Central South University. Written informed consent was obtained from all patients.

### DNA isolation and methylation analysis

#### DNA isolation and bisulfite conversion

DNA extraction from plasma and fresh frozen tissue samples were performed using our previously described MOB approach [18–20], a process that allows DNA extraction and bisulfite conversion in a single tube via the use of silica super magnetic beads. We have optimized the protocol for plasma by adding 4 mL of plasma to 800 μL of proteinase K (800 units/mL, Invitrogen, USA) and 4 mL of Buffer AL (Qiagen, USA), and incubating them together at the same temperature (55 °C overnight). After digestion, 4 mL of isopropyl alcohol (IPA) and 200 μL of beads were added. For DNA extraction from fresh frozen tissue sample, 2–5 mg of tissue sample were added to 40–60 μL of proteinase K and 300 μL of Buffer AL. After incubating overnight, 300 μL of IPA and 150 μL of beads were added. Then, the lysate was incubated and rotated for 10 min before adding 5 μL of carrier RNA, and incubating for an additional 5 min. The bisulfite conversion was performed using a thermal cycler, the optimized incubation temperature and time were programmed as showed in Supplemental Table S2.

#### DNA methylation analysis

The DNA methylation analysis was performed using quantitative real-time methylation-specific PCR and normalized to a control β-Actin (ACTB) assay, as previously described [18]. Amplification reactions were performed using ABI StepOnePlus Real-Time PCR system (Applied Bio.) with all samples being analyzed in triplicate. Thermo cycling conditions were optimized as follows: 95 °C for 5 min, 50 cycles at 95 °C for 15 seconds, and 60 °C for 30 seconds and 72 °C for 30 seconds. The primer and hybridization probe sequences for MOB-qMSP analysis are listed in Supplemental Table S3.

As described previously [18], the 2^−ΔCt^ was calculated for each methylation detection replicate comparing with the mean Ct for ACTB. For replicates which were not detected (ND), a Ct value of 100 was used, creating a near zero value for 2^−ΔCt^. The mean 2^−ΔCt^ value was calculated with the formula:

$$ \mu\ {2}^{-\Delta \mathrm{Ct}}=\frac{\left({2}^{-\Delta  \mathrm{Ct}\ \mathrm{replicate}\ 1}+{2}^{-\Delta  \mathrm{Ct}\ \mathrm{replicate}\ 2}+{2}^{-\Delta  \mathrm{Ct}\ \mathrm{replicate}\ 3}\right)}{3} $$

#### Statistical analysis

Demographic and methylation variables were summarized by case-control status with percentages for categorical variables and means and standard deviations for continuous variables. Differences in demographic variables between cases and controls were assessed with Fisher’s exact test for categorical variables and the Wilcoxon rank sum test for continuous variables. The association between the methylation and case-control status was expressed as odds ratios and their corresponding 95% confidence intervals (CI) obtained from logistic regression models with adjustment for the variables including age, gender, smoking status, pack-years of smoking, COPD, and pulmonary function test results. The pack-years of cigarette smoking were defined as the average number of packs smoked per day times the number of years smoked.

To determine the performance of each individual gene, receiver operating curve (ROC) analysis was performed using the 2^−ΔCt^ values. The area under the curve (AUC) was reported with 95% confidence intervals. On the basis of ROC curves, the three best-performing genes were selected for combined detection in analyses for diagnostic accuracy for lung cancer detection. The best-performing genes as gene panels were also analyzed combined with clinical characteristics [11, 18].

## Results

### Clinical characteristics of the patients

A total of 246 patients met inclusion criteria, with 163 patients with lung cancer and 83 with non-cancerous lung lesions as the benign group. All lesions were no larger than 3.0 cm in greatest dimension verified by pathological reports. All cancer cases were histologically confirmed to be NSCLC with negative lymph nodes. According to the 8th edition of National Comprehensive Cancer Network Guidelines for the TNM classification for lung cancer, all of the NSCLC cases were T_1-2_N_0_M_0_ (stage Ia-Ib) non-small cell lung cancer (some tumors were T2 based on visceral pleural invasion and not size). Clinical and demographic variables showed no differences between the cancer and benign groups except for age (Table 1). Plasma samples of 20 healthy volunteers were also obtained as a normal group (Supplemental Table S1).

### Detection of DNA methylation in plasma and tumor samples

**Fig. 1:**
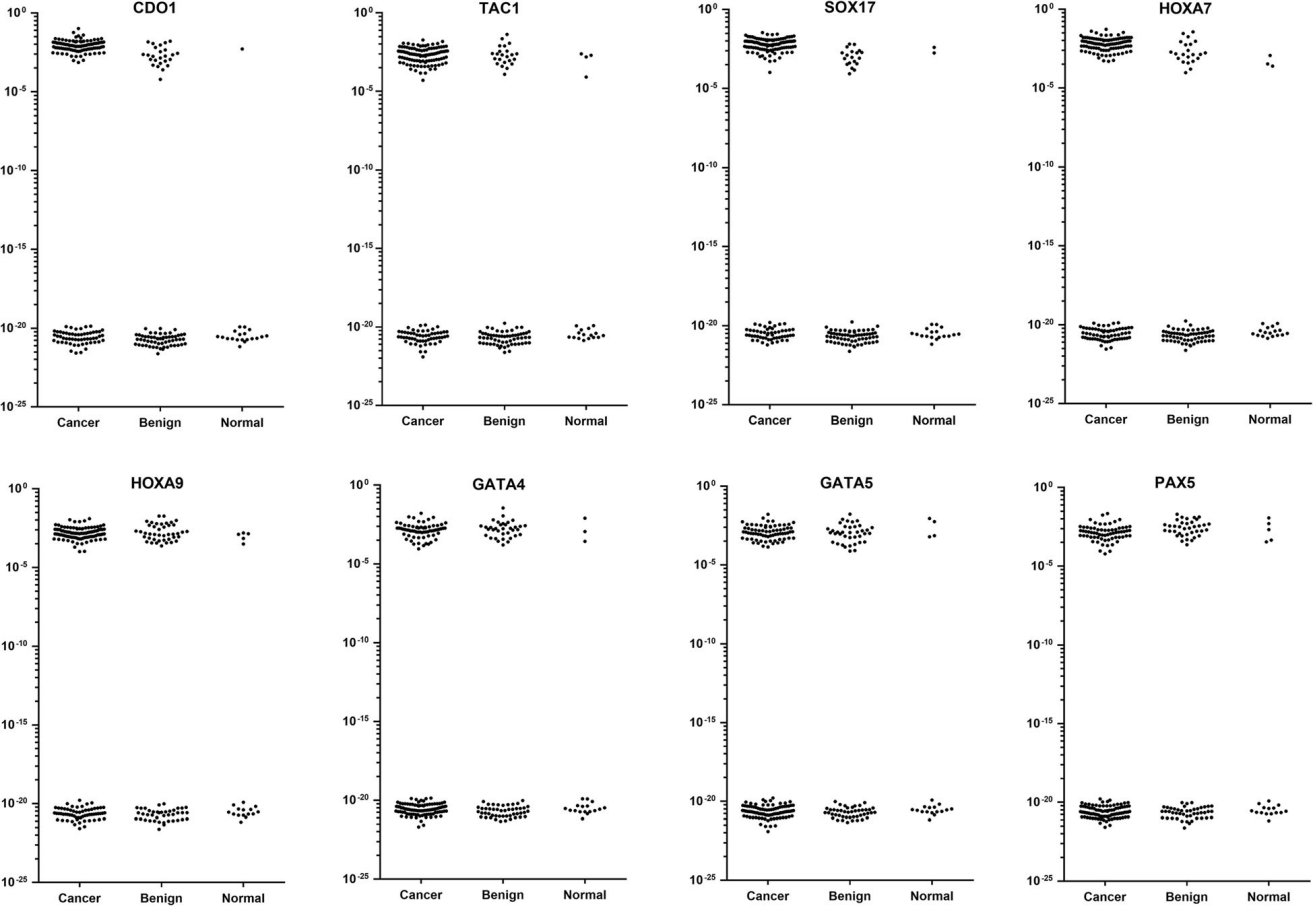
Methylation profiles of the eight genes from plasma samples. This scatter plot shows the converted ΔCt methylation values in a logarithmic scale. These plots show a bimodal distribution with the lower group the values corresponding to those samples with no detectable amplification (ND). Compared with cancer and benign group, the healthy group had the lowest methylation rate in all the 8 genes. The methylation rate of CDO1, TAC1, SOX17, and HOXA7 was significantly higher in cancer group than that in benign group

Methylation of these eight genes from plasma and tumor samples was detected using modified MOB-qMSP approach. In plasma samples, compared with cancer and benign group, the healthy group had the lowest methylation rate in all the eight genes (*p* < 0.001). The methylation detection rate of CDO1, TAC1, SOX17, and HOXA7 were significantly higher in cancer group than in the benign group (*p* < 0.001) (Fig. 1). We first determined the diagnostic sensitivity and specificity according to the presence or absence of detectable methylation, without considering quantitation of DNA methylation (Table 2) [18]. The sensitivity and specificity for lung cancer diagnosis using individual genes from plasma ranged from 41 to 69% and 49 to 82%, respectively, with the best-performing genes being those previously studied. The newly examined genes did not perform as well as these loci. The eight gene methylation status in tumor tissues were also detected using modified MOB-qMSP. Consistent with DNA methylation profiles in plasma, methylation of CDO1, TAC1, SOX17, and HOXA7 were detected more frequently in patients with cancer compared with controls (Supplemental Figure S1).

**Table 2:** Gene methylation detection in plasma samples

Gene	Cancer (*n* = 163)		Control (*n* = 83)			
	*n*	Sensitivity	*n*	Specificity	PPV	NPV
CDO1	106	65%	17	80%	86%	54%
TAC1	110	67%	26	69%	81%	52%
SOX17	113	69%	15	82%	88%	58%
HOXA7	98	60%	15	82%	87%	51%
HOXA9	101	62%	42	49%	71%	40%
GATA4	68	42%	35	58%	66%	34%
GATA5	72	44%	38	54%	65%	33%
PAX5	67	41%	37	55%	64%	32%

### Gene methylation and lung cancer diagnostic accuracy

We then generated ROC curves for each gene using normalized methylation 2^−ΔCT^ calculated as described previously [18]. At the best quantitative cutoff, the sensitivity and specificity for lung cancer diagnosis from each single methylated gene ranged from 41 to 68% and from 49 to 87, respectively (Table 3). These sensitivities and specificities were similar to that obtained from the absolute presence of detectable methylation for each gene (Table 2).

**Table 3:** Sensitivity, specificity, PPV, and NPV at optimal cutoffs with AUC

Gene	Sensitivity	Specificity	PPV	NPV	AUC	95% CI
CDO1	63%	83%	88%	53%	0.78	0.71–0.83
TAC1	68%	70%	81%	52%	0.71	0.64–0.78
SOX17	68%	86%	90%	57%	0.82	0.76–0.87
HOXA7	55%	87%	89%	50%	0.73	0.67–0.80
HOXA9	64%	49%	71%	41%	0.56	0.48–0.64
GATA4	44%	58%	67%	35%	0.53	0.45–0.61
GATA5	43%	63%	70%	36%	0.52	0.44–0.60
PAX5	41%	55%	64%	32%	0.54	0.45–0.62
CDO1, TAC1, SOX17	89%	61%	82%	74%	0.85	0.81–0.91
CDO1, SOX17, HOXA7	90%	71%	86%	78%	0.88	0.84–0.93

**Fig. 2:**
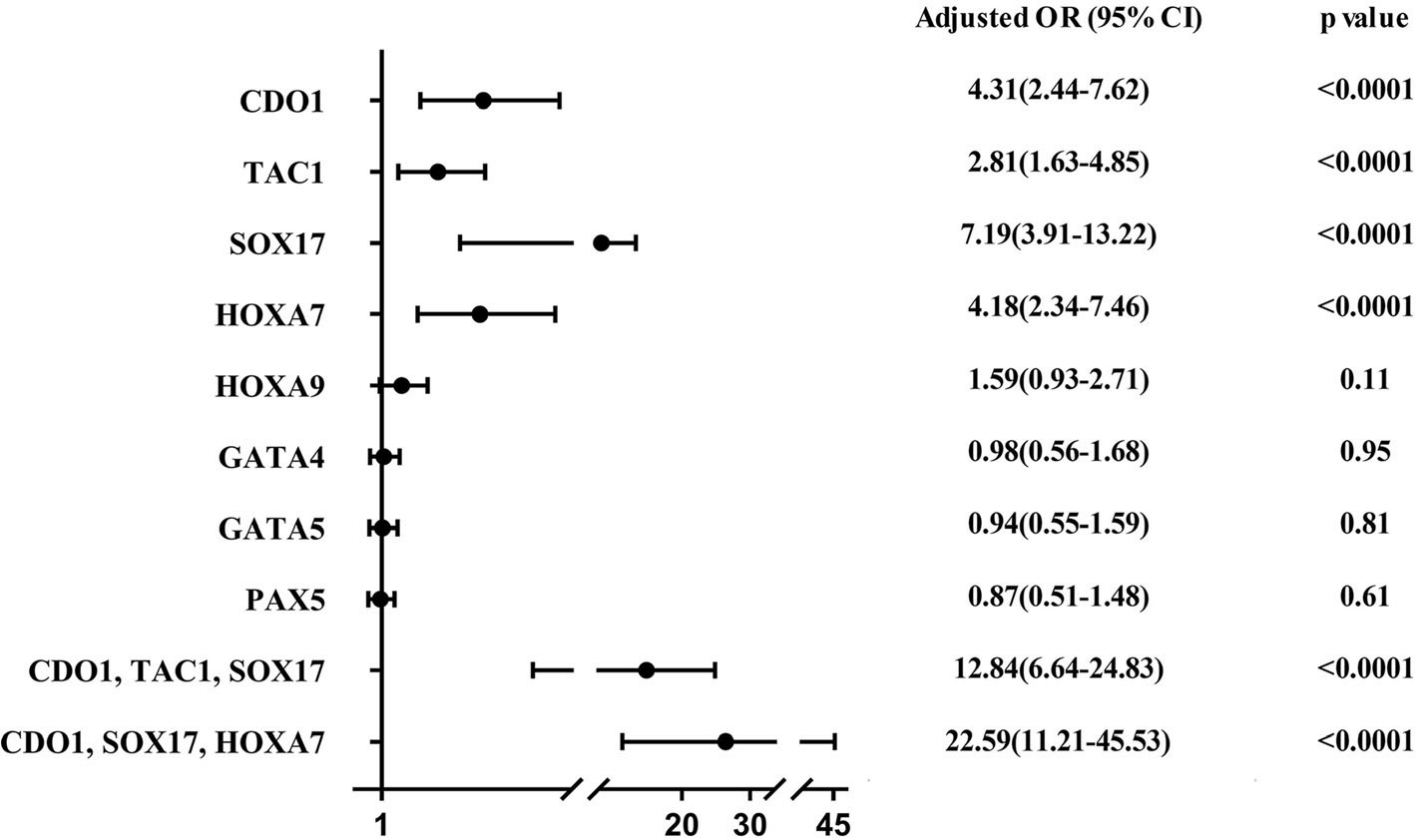
Performance of gene methylation as predictor for lung cancer. The logistic regression analyses indicated that the methylation of CDO1, TAC1, SOX17, and HOXA7 were closely related to increasing of lung cancer risk. With the best adjusted odds ratio, the combination of CDO1, SOX17, and HOXA7 showed the best performance in the diagnosis of lung cancer

The genes with the largest AUC value were as follows: CDO1: AUC 0.78, 95% CI 0.71–0.83; TAC1: AUC 0.71, 95% CI 0.64–0.78; SOX17: AUC 0.82, 95% CI 0.76–0.87; and HOXA7: AUC 0.73, 95% CI 0.67–0.80. The PPV and NPV for these genes were for CDO1, 88% and 53%; for TAC1, 81% and 52%; for SOX17, 90% and 57%; and for HOXA7, 89% and 50%, respectively (Table 3, Supplemental Figure S2). As elevated odds ratios ranged from 2.81 to 7.19, logistic regressions analyses also indicated that the methylation of CDO1, TAC1, SOX17, and HOXA7 were closely related to increasing lung cancer risk (Fig. 2).

**Fig. 3:**
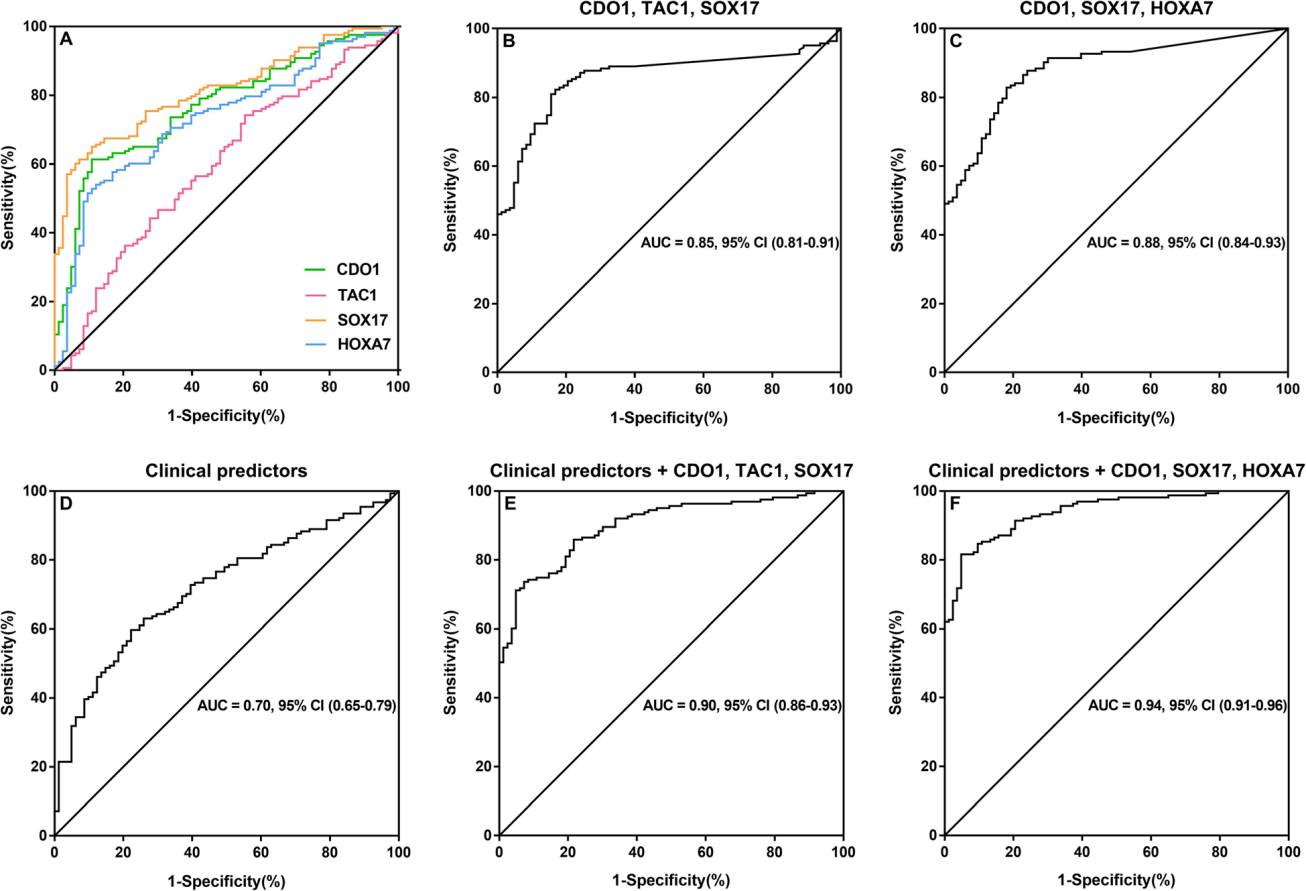
ROC curves for lung cancer detection. **a** ROC curves comparing the four genes with the largest areas under the curve in plasma. **b** ROC of the combined methylation status of CDO1, TAC1, and SOX17 from plasma with the largest area under the curve. **c** ROC of the combined methylation status of CDO1, SOX17, and HOXA7 from plasma with the largest area under the curve. **d**, **e** and **f** ROC curves assessing the accuracy of the predictions for lung cancer using gene methylation panel with clinical risk factors(age, pack-year, COPD status, nodule size, and pulmonary function values). **d** Plot is obtained using clinical predictors alone. **e** Plot is obtained using clinical predictors plus the combination of CDO1, TAC1, and SOX17. **f** Plot is obtained using clinical predictors plus the combination of CDO1, SOX17, and HOXA7

We further evaluated the combination of the three best-performing genes. The combination of CDO1, TAC1, and SOX17, which was evaluated as the best combination in our previous study, was examined in the current Chinese cohort. The sensitivity and specificity were 89% and 61% respectively, with AUC of 0.85 (95% CI, 0.81–0.91). However, different from our previous publication, the combination of best three genes, CDO1, SOX17, and HOXA7, showed the best sensitivity and specificity of 90% and 71%, with a corresponding ROC AUC of 0.88 (95% CI 0.84–0.93; Table 3, Fig. 3a, b, c). This improved performance for this three-gene combination appears to be from a higher prevalence/sensitivity of HOXA7 in this cohort, compared with that previously reported [18], with maintained high specificity. With an adjusted odds ratio of 22.59, (95% CI 11.21–45.53), the combination of CDO1, SOX17, and HOXA7 showed the best association with the diagnosis of early stage NSCLC (Fig. 2). Furthermore, we investigated clinical features (age, pack-year, chronic obstructive pulmonary disease (COPD) status, nodule size, and pulmonary function values) for diagnostic accuracy as clinical predictors alone and in combination with gene panels. Clinical predictors alone had a diagnostic accuracy AUC of 0.70 (95% CI, 0.65–0.79) (Fig. 3d). The best-gene panel combined with the clinical predictors improved the diagnostic accuracy with an AUC of 0.94 (95% CI, 0.91-0.96), slightly better than CDO1, TAC1, and SOX17 plus clinical predictors (Table 4, Fig. 3d, e, f).

**Table 4:** Performance of gene methylation panel in the prediction of early stage lung cancer

Gene	Sensitivity	Specificity	PPV	NPV	AUC	95% CI
Clinical predictors alone	81%	47%	75%	56%	0.70	0.65–0.79
Clinical predictors + CDO1, TAC1, SOX17	93%	63%	83%	82%	0.90	0.86–0.93
Clinical Predictors + CDO1, SOX17, HOXA7	91%	79%	89%	82%	0.94	0.91–0.96

*AUC*, area under the curve (in the ROC curves); *95% CI*, 95 % confidence interval

### Diagnostic accuracy according to tumor size

As the quantity of circulating tumor DNA for detection is related to stage and tumor volume [22], we explored the diagnostic accuracy of the combination of best three genes (CDO1, SOX17, and HOXA7) according to tumor size. In tumors with greatest diameter of 2.1–3 cm, the sensitivity and specificity were 91% and 90%, with the AUC of 0.95, (95% CI 0.90–1.00); in 1.1–2.0 cm tumors, the sensitivity and specificity were 74% and 93%, and the AUC was 0.92, (95% CI 0.87–0.96); in tumors less than 1.0 cm, the sensitivity and specificity decreased slightly to 64% and 82%, with a corresponding AUC of 0.75, (95% CI 0.62–0.89) (Tables 5, 6 and 7). Interestingly, while the combination of CDO1, SOX17, and HOXA7 showed the overall best performance in our patient cohort, the combination of CDO1, TAC1, and SOX17 had the best performance in the subgroup with tumor size less than 1.0 cm (Tables 5, 6 and 7).

**Table 5:** Sensitivity, specificity, PPV, and NPV at optimal cutoffs with AUC regarding tumor size (2.1–3 cm)

Gene	Sensitivity	Specificity	PPV	NPV	AUC	95% CI
CDO1	67%	89%	94%	53%	0.77	0.64–0.90
TAC1	65%	72%	85%	46%	0.67	0.51–0.83
SOX17	67%	90%	94%	53%	0.79	0.68–0.90
HOXA7	70%	83%	91%	54%	0.77	0.65–0.89
CDO1, TAC1, SOX17	88%	89%	95%	76%	0.91	0.83–0.99
CDO1, SOX17, HOXA7	91%	90%	96%	81%	0.95	0.90–1.00

Cancer, *n* = 43; control, *n* = 18

**Table 6:** Sensitivity, Specificity, PPV, and NPV at optimal cutoffs with AUC regarding tumor size (1.1–2 cm)

Gene	Sensitivity	Specificity	PPV	NPV	AUC	95% CI
CDO1	72%	88%	93%	59%	0.80	0.73–0.88
TAC1	70%	70%	83%	52%	0.69	0.60–0.79
SOX17	65%	93%	95%	55%	0.82	0.75–0.89
HOXA7	61%	77%	85%	48%	0.72	0.63–0.81
CDO1, TAC1, SOX17	73%	93%	96%	62%	0.91	0.87–0.97
CDO1, SOX17, HOXA7	74%	93%	96%	63%	0.92	0.87–0.96

Cancer, *n* = 92; control, *n* = 43

**Table 7:** Sensitivity, Specificity, PPV, and NPV at optimal cutoffs with AUC regarding tumor size (0–1 cm)

Gene	Sensitivity	Specificity	PPV	NPV	AUC	95% CI
CDO1	82%	46%	66%	67%	0.64	0.49–0.80
TAC1	61%	78%	78%	61%	0.68	0.56–0.81
SOX17	61%	77%	77%	61%	0.68	0.56–0.80
HOXA7	82%	59%	72%	72%	0.73	0.60–0.87
CDO1, TAC1, SOX17	71%	82%	83%	69%	0.81	0.69–0.93
CDO1, SOX17, HOXA7	64%	82%	82%	64%	0.75	0.62–0.89

Cancer, *n* = 28; control, *n* = 22

## Discussion

In this study, using modified MOB-qMSP, we investigated the detection of promoter hypermethylation of eight genes and one internal control gene in plasma and tumor samples of patients with small lung nodules. This study is a corroboration of our previous study [18], but now examined in a Chinese cohort, suggesting that these detection biomarkers are useful in divergent populations. Although our previous study had demonstrated the high diagnostic sensitivity and specificity of promotor methylation of CDO1, TAC1, HOXA7, HOXA9, and SOX17 in plasma from patients with NSCLC in a Lung Cancer Specialized Program of Research Excellence (SPORE) patient cohort [18, 23], the performance and diagnostic accuracy of these biomarkers still needed validation in another cohort, and might be affected by differences between races, environmental carcinogenic exposure, and smoking status.

In the present study, we evaluated the performance of individual gene biomarkers for the early detection of lung cancer (Tables 2 and 3). This confirmed the utility of CDO1, TAC1, HOXA7, and SOX17, while newly tested genes were not as effective for lung cancer detection. While each gene can detect lung tumor DNA from many patients, given the rarity of these molecules in ctDNA, individual gene sensitivity is somewhat limited. However, by combining the best-performing genes in a panel, we greatly improve the diagnostic sensitivity without a substantial decline in specificity. From the initial screening of eight genes, four genes were selected as candidates for a panel to distinguish early stage NSCLC from benign lung nodules, which had previously also provided the best cancer sensitivity and specificity. The possible 3 gene combinations (CDO1, TAC1, and SOX17; or CDO1, SOX17, and HOXA7) were able to provide a very high sensitivity ranging from 89 to 90% and a specificity ranging from 61 to 71%. Due to the fact that the variations of DNA methylation patterns in individuals might depend on the alterations of different molecular pathways, the use of a multiple gene panel may provide greater utility for detecting different tumors when compared with a single gene [24]. However, the primary benefit of multigene detection is likely from the additional chances this allows to detect rare ctDNA molecules in plasma samples.

ctDNA in plasma can carry abnormal cellular alterations related to cancer. Several studies have sought to improve early detection of lung cancer by investigating molecular biomarkers in the plasma [25, 26]. However, none of these tests have been widely used in practice due to unsatisfactory sensitivities and specificities. Our previous study demonstrated the high diagnostic accuracy for early-stage lung cancer using a panel of methylated promoter genes in the plasma based on MOB-qMSP strategy [18]. The promoter hypermethylation of CDO1, TAC1, HOXA7, and SOX17 were detected more frequently in the plasma of cancer patients compared with controls. The combination of CDO1, TAC1, and SOX17 showed the highest sensitivity and specificity (93% and 62%). In the present study, higher methylation frequencies of CDO1, TAC1, HOXA7, and SOX17were also observed in the plasma of cancer patients. The combination of CDO1, TAC1, and SOX17 showed similar sensitivity and specificity (89% and 61%) which was consistent with our previous results. However, differing from our previous study, the combination of CDO1, SOX17, and HOXA7 showed even better sensitivity and specificity (90% and 71%) in the Chinese cohort, indicating better diagnostic accuracy, which appears to be from the higher prevalence of HOXA7 methylation detection in this population. The performance of these gene methylation markers remains their superiority in differentiating lung cancer from benign lung nodules, suggesting the potential clinical application for the diagnosis of early stage lung cancer.

Capitalizing on the strengths of highly prevalent DNA methylation biomarkers and ultra-sensitive techniques to detect DNA methylation could facilitate early diagnosis of lung cancer with indeterminate screen-detected pulmonary nodules [27, 28]. Recently, Liang and colleagues reported the high diagnostic accuracy by using high-throughput DNA bisulfite sequencing in tissue and plasma samples from patients with lung cancer [29]. Other studies have also sought to differentiate lung cancers from benign lung nodules by investigating ctDNA markers [30–32]. However, these studies mainly used next-generation sequencing technologies which are more costly due to the depth of sequencing required and also needs extensive bioinformatics analyses, which reduce the ease of clinical application. Easy, efficient, and cost-effective detection of regional DNA methylation could reduce testing costs and has enormous potential clinical application.

In our series of studies, we employed a newly developed method, methylation on beads (MOB), which permits DNA extraction and bisulfite conversion in a single-tube cellular processing by using superparamagnetic nano-beads as a DNA carrier. As previously described, it yields on average 1.5- to 5-fold improvement in extraction and conversion efficiency compared with traditional column-based technique, with an even greater improvement in detection sensitivity [19, 20, 33]. Followed by real-time quantitative methylation-specific PCR (qMSP), DNA methylation signals from body fluids, such as plasma and sputum, could be easily and efficiently detected by this assay [18]. In the present study, we slightly modified this MOB-qMSP protocol, by shortening the bisulfite converting time but at a higher reaction temperature (Supplemental Table S2), which could minimize DNA damage during bisulfite conversion and increase the efficiency of the detection of ctDNA methylation.

The demographic characteristics of the cancer group and control group in this study differed slightly (Table 1). Similar to our previous study, methylation detection of these genes was not associated with gender, smoking status, and pulmonary function. One factor that might relate to diagnostic accuracy was tumor size. Several studies have reported lower detection rates with decreasing tumor size, especially for biomarkers detected in plasma [14, 34]. In the previous study based on mainly American patients, the diagnostic accuracy of single gene or gene panels of combined genes showed no differences among subgroups of different sizes, but few tumors were sub-centimeter in size. In the present study, the gene panel CDO1, SOX17, and HOXA7 showed the best diagnostic accuracy in the subgroup with tumor size 2.1-3 cm and decreased in subgroups with smaller tumor size, especially when the tumor size was less than 1 cm. On the other hand, in the subgroup with tumor size 0–1 cm, the gene panel CDO1, TAC1, and SOX17 (sensitivity 71%, specificity 82%, and AUC 0.81, respectively) was slightly better than gene panel CDO1, SOX17, and HOXA7 (64%, 82%, and 0.75, respectively), indicating this panel may have better sensitivity and diagnostic accuracy in the detection of very small lung cancer lesions (Tables 5, 6 and 7). While the sensitivity for tumors < 1 cm is slightly lower than larger tumors, previous studies have not been able to detect this tumors even this well, and in most cases did not attempt to detect such early-stage lung cancer [29, 35, 36].

Promoter region hypermethylation is an important mechanism of gene silencing involved in several physiological and pathological processes, especially in the initiation and progression of cancer [37, 38]. Several studies have reported that the presence of promoter hypermethylation of tumor suppressor genes could be observed in control populations as a random or a physiologic event related to age, smoking status, or environmental carcinogenic exposures, which decreased the diagnostic accuracy of cancer when using DNA methylation as a biomarker [39, 40]. This consideration brings up the necessity of validation of the performance of DNA methylation biomarkers within different populations, and emphasizes the importance that the control group should be of similar age and exposure. While in the present study, DNA methylation of single genes could be detected in plasma of some patients with benign lung nodules, and more rarely in healthy controls, there remains a high sensitivity and specificity in this screen-detected lung nodule cohort. This suggests this approach has diagnostic accuracy and suggests the potential for clinical application in the evaluation of screen-detected nodules.

The National Lung Screening Trial (NLST) reported a sensitivity of 93% in baseline scans but a false positive rate of 26.3% (specificity of 74%) and a false discovery rate of 96%. This would make our plasma-based detection to have a similar performance to CT detection. However, a direct comparison is not possible, since we only examined nodules < 3 cm, and it is likely our sensitivity would be greater if larger nodules were included. The primary utility would not be an alternative to CT screening, but as a complimentary test to enhance the management of detected nodules. With this level of sensitivity and specificity, a simple non-invasive test could potentially reduce the need for invasive confirmation tests needed to establish or rule out the diagnosis of cancer in patients with indeterminate pulmonary nodules detected by CT screening.

## Conclusion

Taken together, this study demonstrates that, with modified MOB-qMSP assay, detection of early stage NSCLC with high sensitivity and specificity could be obtained using a panel of methylated promoter genes in plasma, even extending this detection to sub-centimeter nodules. This gene panel and detection strategy have great potential for an adjunct to CT screening, identifying patients at high risk for lung cancer, reducing false positive results, and improving the diagnosis of lung cancer at an earlier stage.

## Supplementary information

**Additional file 1.** Supplemental Figure S1. Methylation profiles of the 8 genes from tissue samples. Consistent with DNA methylation profiles in plasma, methylation of CDO1, TAC1, SOX17, and HOXA7 were detected more frequently in patients with cancer compared with benign controls. Supplemental Figure S2. Receiver operator classification curves for lung cancer detection for the 8 genes obtained from Plasma. Supplemental Table S2. Bisulfite conversion thermal cycler conditions. Supplementary Table S3. Primers and probes of qMSP for plasma and tissue samples.

## Data Availability

The datasets used and/or analyzed during the current study are available from the corresponding author on reasonable request.
