# Supplementary material for: Ultrasensitive DNA hypermethylation detection using plasma for early detection of NSCLC: a study in Chinese patients with very small nodules
Source: Clin Epigenetics. 2020 Mar 5;12:39. doi: 10.1186/s13148-020-00828-2 (PMC7057485; doi:10.1186/s13148-020-00828-2)
Supplement: Supplementary file 1 — Additional file 1. Supplemental Figure S1. Methylation profiles of the 8 genes from tissue samples. Consistent with DNA methylation profiles in plasma, methylation of CDO1, TAC1, SOX17, and HOXA7 were detected more frequently in patients with cancer compared with benign controls. Supplemental Figure S2. Receiver operator classification curves for lung cancer detection for the 8 genes obtained from Plasma. Supplemental Table S2. Bisulfite conversion thermal cycler conditions. Supplementary Table S3. Primers and probes of qMSP for plasma and tissue samples. [file 13148_2020_828_MOESM1_ESM.docx]

Supplemental Figure S1.

Methylation profiles of the 8 genes from tissue samples. Consistent with DNA methylation profiles in plasma, methylation of CDO1, TAC1, SOX17, and HOXA7 were detected more frequently in patients with cancer compared with benign controls.


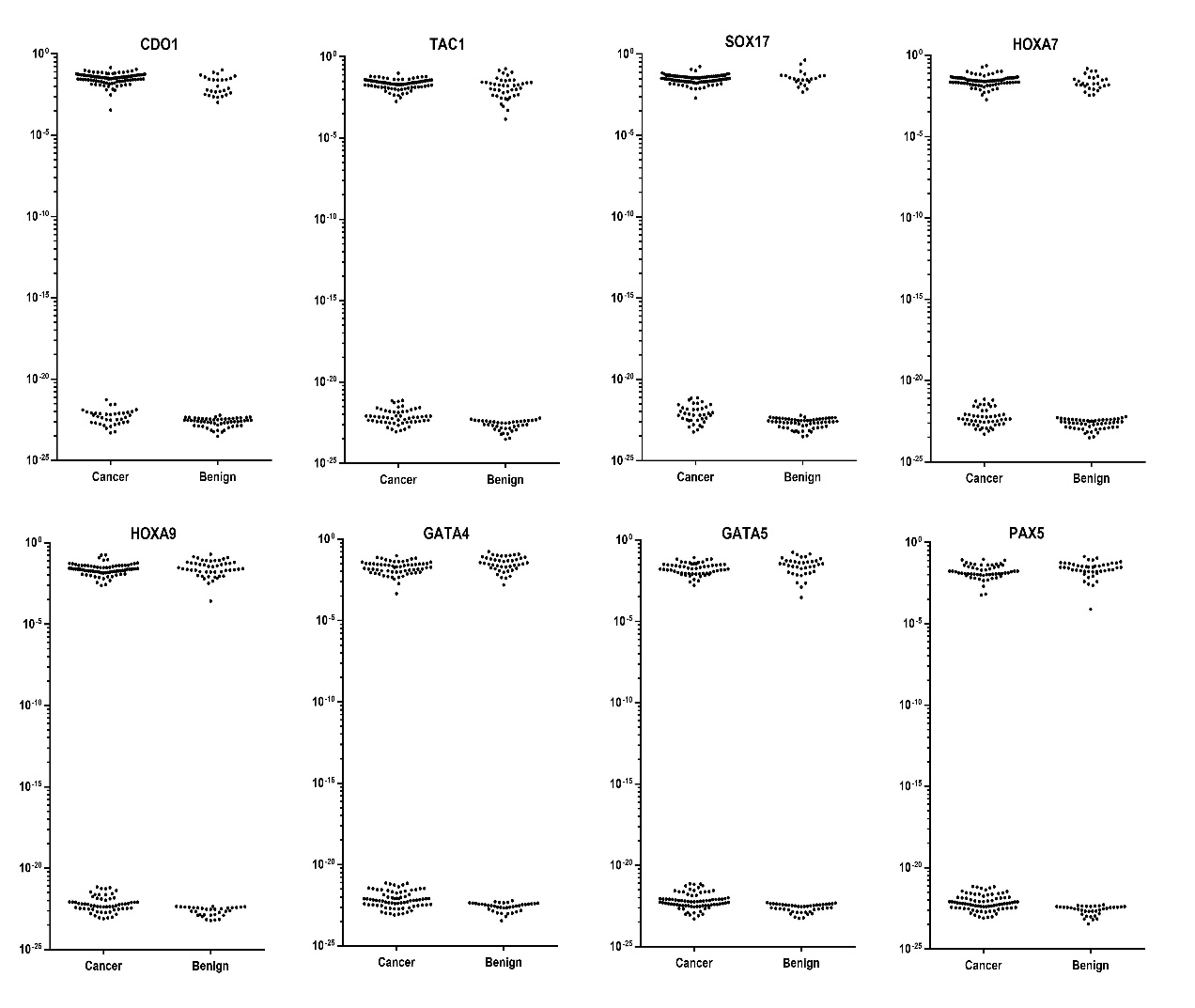


Supplemental Figure S2.

Receiver operator classification curves for lung cancer detection for the 8 genes obtained from Plasma.


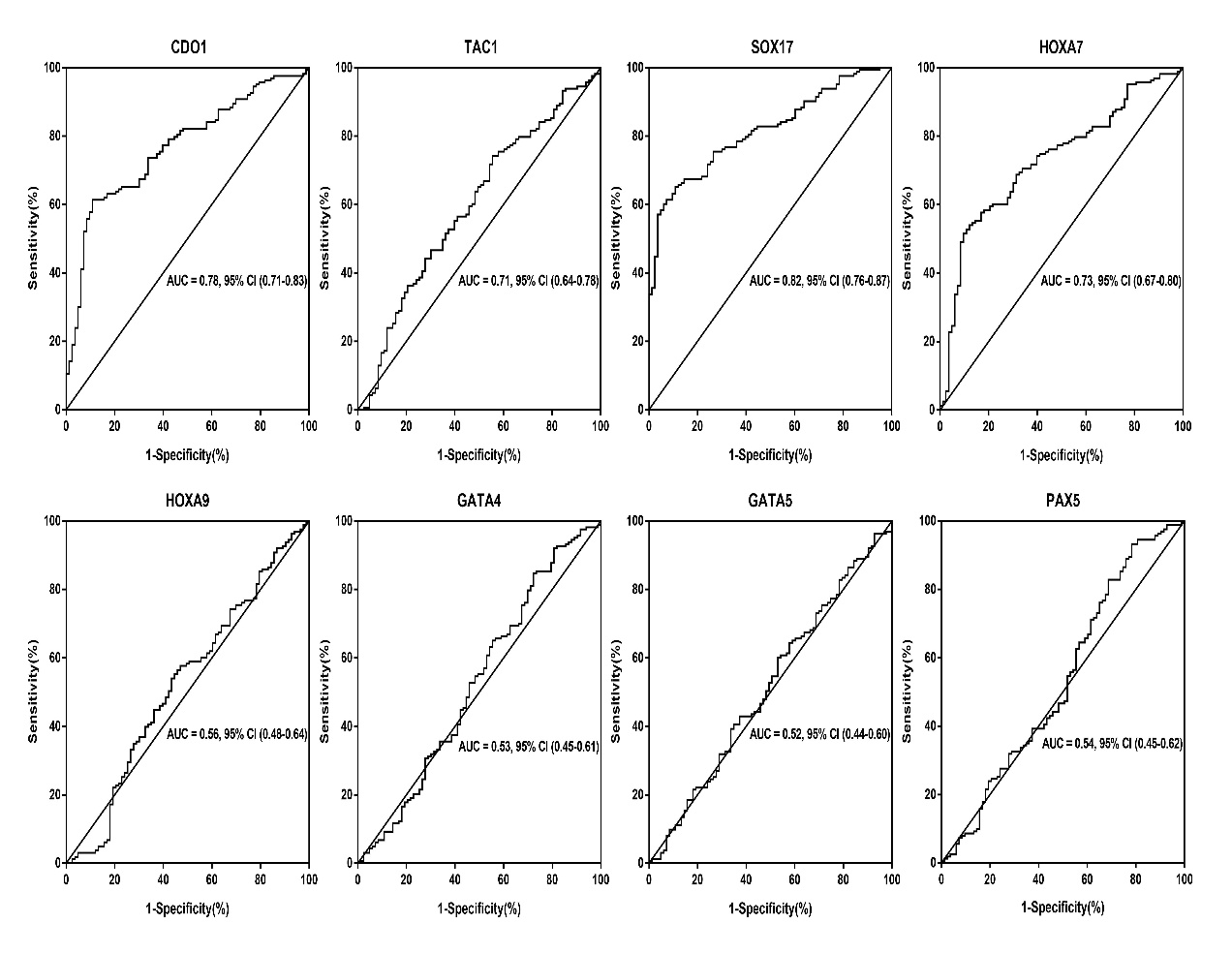


Supplemental Table S1. Clinical characteristics of healthy volunteers.

| Clinical characteristics | Healthy volunteers (n=20) |
| --- | --- |
| Age (year, mean ± SD) | 51.15±5.35 |
| Gender-no. (%) |  |
| Male | 9 (45%) |
| Female | 11 (55%) |
| Pack-Year (IQR) | 37.5 (5-60) |
| COPD-no. (%) | 0 |

*Abbreviations*: COPD, Chronic obstructive pulmonary disease; IQR, Interquartile range.

Supplemental Table S2. Bisulfite conversion thermal cycler conditions.

| Steps | Time | Temperature |
| --- | --- | --- |
| Denaturation | 5 min | 95°C |
| Incubation | 30 min | 60°C |
| Denaturation | 5 min | 95°C |
| Incubation | 90 min | 60°C |
| Denaturation | 5 min | 95°C |
| Incubation | 150 min | 60°C |
| Hold | No longer than 12h | 4°C |

### Supplementary Table S3. Primers and probes of qMSP for plasma and tissue samples.

| **Gene** | **Forward 5' - 3'** | **Reverse 5' - 3'** | **Probe** | **Size (bp)** | **Tm (℃)** |
| --- | --- | --- | --- | --- | --- |
| CDO1 | CGGAGGCGGGGAGATTTTGCG | CGAAACCCACGCGATCCCTAAAACG | TTTCGTTGTTTTCGGCGTTTTAGGGATCGCGTGGG | 116 | 60 |
| TAC1 | CGGTTAATTAAATATTGAGTAGAAAGTCGCG | CTCCGCACTCTCGATAACTACCG | GGAGAATGTTACGTGGGTTTGGAGGTTTAAGGAGG | 153 | 60 |
| SOX17 | TTGGATTGGGACGTGGGATTCG | GAAAACGAACCGATCCCGCG | GTGGGTTTAACGACGCGGGATCGGTTCG | 70 | 60 |
| HOXA7 | GTGGTTTCGTTTCGTAGGGTTCG | CCGCCAACTAAACGCTCCC | GCGGAAAAAGATTTGGAGGTTTCGCGGGAG | 148 | 60 |
| HOXA9 | AGTTATATAGGTTGGCGGGAAGTCG | TCGCCTCTCCCGAAAACC | AGGTTAGATTGTTGTGTTTGGTTGGCGAGTT | 160 | 60 |
| GATA4 | TCGTATAGTTTCGTAGTTTGCGT | ACTCGACCCTAAAACGCTAAC | TAGTCGGGGTCGCGTATTTT | 109 | 60 |
| GATA5 | AATCGCGGGGTTTTCGTTAG | AACACGCGACGATAAAACCC | GGTTACGTAACGAGGGGGTT | 140 | 60 |
| PAX5 | GCGTAAGAGAGACGAAGGTAAG | ATATTCGCGAACACCTCTACTAC | AGAGGTTCGCGTAGTTTCGTCGG | 112 | 60 |
| ACTB | TAGGGAGTATATAGGTTGGGGAAGTT | AACACACAATAACAAACACAAATTCAC | TGTGGGGTGGTGATGGAGGAGGTTTAG | 103 | 60 |
